# Association of Funding Cuts to the Patient Protection and Affordable Care Act Navigator Program With Privately Sponsored Television Advertising

**DOI:** 10.1001/jamanetworkopen.2022.24651

**Published:** 2022-08-01

**Authors:** Rebecca Myerson, David M. Anderson, Laura M. Baum, Erika Franklin Fowler, Sarah E. Gollust, Paul R. Shafer

**Affiliations:** 1 Department of Population Health Sciences, University of Wisconsin–Madison, Madison; 2 Duke Margolis Center for Health Policy, Duke University, Durham, North Carolina; 3 Department of Population Health Sciences, Duke University, Durham, North Carolina; 4 Wesleyan Media Project, Wesleyan University, Middletown, Connecticut; 5 Department of Government, Wesleyan University, Middletown, Connecticut; 6 Division of Health Policy and Management, School of Public Health, University of Minnesota, Minneapolis; 7 Department of Health Law, Policy, and Management, Boston University, Boston, Massachusetts

## Abstract

**Importance:**

Many individuals eligible for coverage in the Patient Protection and Affordable Care Act (ACA) marketplace remain unenrolled because of information barriers. Whether the private sector or the public sector should conduct outreach to address these barriers is a topic of active debate.

**Objective:**

To determine whether cuts to the funding of the ACA navigator program were associated with changes in the volume of private sector advertising.

**Design, Setting, and Participants:**

Using data from the 2015 to 2019 open enrollment periods, this economic evaluation analyzed the changes in advertising associated with 2017 to 2019 cuts to navigator program funding. A difference-in-difference analysis was used to compare outcomes before and after the cuts in counties with higher and lower exposure to the navigator program. Health insurance advertising was measured using data from Kantar/Campaign Media Analysis Group in collaboration with the Wesleyan Media Project, the most comprehensive data available on local broadcast and national cable advertising. The data set included all counties that met the eligibility criteria for the navigator program from 2015 through 2019. Data were analyzed from August 2021 to May 2022.

**Exposures:**

Counties were classified as having higher or lower exposure to the navigator program according to the intensity of program activity in 2016, before the funding cuts. Counties served only by statewide navigator programs were categorized as lower exposure, while those also served by local navigator programs were categorized as higher exposure.

**Main Outcomes and Measures:**

Number of privately sponsored television advertisement airings for the ACA individual health insurance marketplace during the 2015 to 2019 open enrollment periods in each county, adjusted for population.

**Results:**

All counties in 33 states that met the eligibility criteria for the navigator program from 2015 through 2019 were included in the analysis (2435 counties). Cuts to the navigator program were not associated with changes in the number of privately sponsored health insurance advertisements aired. Results were similar under several alternative approaches including an event study specification.

**Conclusions and Relevance:**

In this study of the association between television advertising and navigator funding in the ACA marketplaces, private sector entities did not increase their advertising to compensate for declines in government-sponsored navigator activity. This finding can inform policy debates about the extent to which the private sector adjusts in response to changes in government outreach, and thus improve the design of state waivers and federal funding allocations.

## Key Points

**Question:**

What is the association between the 80% cut in funding for the Patient Protection and Affordable Care Act (ACA) navigator program between 2017 and 2019 and private sector advertising in the ACA individual health insurance marketplace?

**Findings:**

This economic evaluation of 2435 counties in 33 US states using a difference-in-difference analysis found no significant change in the number of private sector advertisements aired targeting marketplace health insurance or other non-Medicare, non-Medicaid health insurance associated with the funding cuts.

**Meaning:**

These findings suggest cuts to funding for the ACA navigator program were not associated with changes in the number of advertisements aired by health insurance companies or other private sector sponsors; the findings can inform policy debates about the extent to which the private sector adjusts in response to changes in government outreach.

## Teaser

This economic evaluation investigates the association between cuts to the Patient Protection and Affordable Care Act (ACA) navigator program and private sector television advertising in the health insurance marketplace.

## Introduction

The Patient Protection and Affordable Care Act’s (ACA) individual health insurance marketplace has insured millions of individuals since 2014. The federally facilitated health insurance marketplace, known as HealthCare.gov, is a public-private partnership that provides a clearinghouse exchange where private insurers offer regulated health insurance plans to individuals. However, barriers to marketplace coverage persist; in 2021, an estimated 12 million people remained uninsured despite being eligible for marketplace coverage, including 6 million who could have received free coverage.^1^ The respective roles that the public and private sectors should play in reducing the remaining barriers to marketplace coverage is a matter of active policy debate.^2^

A lack of awareness about relevant options may be a barrier to gaining coverage. In 2014, nearly two-thirds of uninsured adults reported being exposed to little or no information about financial assistance for marketplace coverage.^3^ Media coverage of the ACA rarely referenced the available financial assistance.^4^ In 2018, there remained an estimated 30 million uninsured people, and two-thirds of uninsured adults had not visited the marketplace to see their coverage options.^5^ Furthermore, many uninsured adults are not familiar with key health insurance terms, such as *deductible* and *premium*.^6,7,8^ These gaps in health insurance literacy are important because plans in the ACA marketplace vary widely in terms of benefits, networks, and premiums.^9,10^ Complex choices and the large number of available options increase the difficulty of selecting a marketplace plan.^11,12,13,14,15^

To address these information barriers and increase marketplace coverage, public and private sector entities have conducted outreach ranging from advertising^16,17^ to direct enrollment assistance.^18^ Since 2014, each state was required to establish assister programs providing free, impartial, one-on-one advice and enrollment assistance.^19^ During the first 3 years these programs were available, more than 28 000 assisters aided more than 20 million potential enrollees.^20,21,22^ In states that used the federally facilitated marketplace, these required public sector assisters were funded via navigator grants from the federal government.^23^

During the Trump administration, public sector outreach efforts changed in several ways relevant to marketplace enrollment.^16,24,25,26^ After the inauguration, the Trump administration canceled the television and radio advertising scheduled for the final week of the 2017 open enrollment period.^27^ Just before the beginning of the 2018 open enrollment period, the administration eliminated all federal funding for television advertising for the marketplace and reduced marketing funding by 90%.^28^ In addition, the Centers for Medicare & Medicaid Services (CMS) unexpectedly cut navigator grants by approximately 40% in 2017.^29^ The following year, CMS decreased navigator program funding even further, resulting in an approximately 80% decline between 2016 and 2018; this reduced funding level remained consistent for the next 2 years.^30,31,32^ Cross-county comparisons suggest that these cuts in funding for local navigator programs substantially reduced coverage among many groups, including individuals with low incomes, those younger than 45 years, those with low English proficiency, and individuals who identify as Hispanic.^25^

Identifying the association between navigator funding and private sector advertising is important for informing future marketplace design, navigator assistance funding, and public sector advertising allocations. In a precaution adoption process model for insurance enrollment, individuals proceed from being unaware, to aware but unengaged, to deciding to enroll or not enroll in coverage, to acting on their decision about enrollment.^33^ Navigators and advertising both plausibly increase the awareness and potential engagement of individuals, while navigators can also aid in navigating the process of choice and enrollment. Thus, for insurers seeking to raise profits by enrolling more people in marketplace coverage, advertising may be a substitute for public funding of the navigator program, albeit an imperfect one. Despite evidence of the association between outreach and health insurance enrollment, including direct enrollment assistance via navigators^25,34,35,36^ and television advertising,^16,24,37,38,39,40^ little research has explored the interplay between public and private sector sources of outreach. To assess the repercussions of recent developments and inform policy decisions, this study examined the association between the Trump-era cuts in navigator program funding and the volume of private sector advertising.

## Methods

### Study Design

This economic evaluation study was deemed exempt from review by the University of Wisconsin-Madison institutional review board because it did not involve human participants and was exempt from the need for informed consent in accordance with 45 CFR §46. We followed the Strengthening the Reporting of Observational Studies in Epidemiology (STROBE) reporting guideline.^41^ We hypothesized that cuts in navigator funding would lead to an increase in private sector health insurance advertising as a compensatory response. We leveraged the approximately 80% reduction in funding for navigator programs between 2017 and 2019 under the Trump administration as a natural experiment (eFigure 1 in the Supplement). In each state, some counties were more exposed to the cuts than others because they were more intensively served by the program before the cuts. For example, counties that were not served by the navigator program at all in 2016 were unaffected by cuts to the program in 2017. We did not use the size of cuts to specific navigator programs as the exposure of interest because the size of funding cuts may have been based on enrollment, an outcome of interest to advertisers.^29^

Following Myerson et al,^25^ we categorized counties as having higher vs lower baseline exposure to the navigator program (and thus to the funding cuts) according to the service areas of navigator grantees in 2016. Counties not served by any navigator program or served only by a statewide program (eg, the Affiliated Service Providers of Indiana) were classified as lower exposure, while counties served by both statewide and nonstatewide (hereafter, local) navigator programs (eg, Community Action of Southern Indiana) were classified as higher exposure.

We used difference-in-differences models to estimate the association between navigator program funding cuts and insurers’ advertising decisions. The first difference is the change in advertising volume before and after the funding cuts. The second difference is the relative change in advertising volume in lower exposure and higher exposure counties. This design allowed us to control for concurrent state-level and national-level policy changes that may influence the results. Advertising specifically targeted to Medicare, a health insurance market not directly affected by cuts to the navigator program, was included as a placebo test. For further confidence in the robustness of our approach, we performed several event studies with varying specifications.

### Data

The primary outcome was the number of privately sponsored non-Medicare and non-Medicaid health insurance television advertisements airings during the 2015 to 2019 open enrollment periods in each county. Kantar/Campaign Media Analysis Group supplied the Wesleyan Media Project with the airing-level advertising data, including information on the media market in which each advertisement aired as well as the date, time, and sponsor of the advertisement. We used a method described in previous work to categorize advertisements by whether they were privately sponsored and by product category (Medicare, Medicaid, and private non-Medicare, non-Medicaid; the final category included marketplace, non–ACA-compliant plans, and employer-sponsored coverage).^16^ In addition, advertisements were categorized according to the open enrollment period in which they aired. Open enrollment periods begin in the autumn before the plan year, which starts on January 1, and end either in the December before the plan year or at some point early in the plan year.^42^ The advertising data from each open enrollment period were crosswalked from the media market level to the county level by assigning each county to a single media market in which it had the largest share of its population because all areas within a given media market are exposed to the same television advertising; this method assigns all counties in the market to the same advertising volume. We excluded advertisements that could not be categorized (0.4%). Interrater reliability was high for Medicare coding (κ = 0.85) and Medicaid coding (κ = 0.7).

The main variable was the county’s exposure to the navigator program in 2016. To classify counties as higher exposure or lower exposure, we used publicly available CMS records that detail which counties were served by each navigator organization.^43^ Detailed records are available because CMS funds the navigator program via grants to specific organizations, rather than grants to states. Additional details on the classification method are available in prior work.^25^

The analytical data set included counties in the 33 states that met the eligibility criteria for the navigator program throughout the study period of 2015 to 2019 (ie, did not have a state-based marketplace in any year between 2015 and 2019). As a result of this exclusion criterion, all states in the analytical sample were observed for the same number of periods both before and after funding cuts.

### Statistical Analysis

To identify the association between the cuts to the navigator program and advertising outcomes, we used county-level differences in prior navigator funding within each state as the source of identification. The first cuts to the navigator program were announced and implemented in the summer of calendar year 2017, just before the open enrollment period for 2018. Accordingly, the 2018 open enrollment period was the start of the postcut period in the primary analysis. All regressions included state-by–open enrollment period and county indicator variables to control for time-invariant differences across counties, other state-level and national-level policy changes, and secular trends unrelated to the funding cuts.^44^ Regressions controlled for key determinants of advertising strategy that might vary within counties over time, including the current population younger than 65 years, the benchmark premium, the spread between the benchmark premium and the least expensive silver plan for a single 40-year old, and the number of carriers participating in the marketplace (because competition and prices may be associated with purchasing decisions).^45,46,47,48^

Heteroskedasticity-robust SEs were clustered by state and treatment group to account for correlation of the error terms according to exposure to the navigator program.^30^ In all analyses, 2-sided *t *tests were used, and a significance level of *P* < .05 was used to determine statistical significance. The eAppendix in the Supplement provides additional details. Data were analyzed using Stata statistical software version 16.1 (StataCorp). Data were analyzed from August 2021 to May 2022.

We conducted several additional sensitivity analyses to assess the validity of the methodological approach and results. The research design assumes that outcomes would have been similar in higher-exposure and lower-exposure counties if there had been no reduction in navigator funding. We conducted event study models to test for violations of this parallel trends assumption.

We assessed the sensitivity of the results to several alternate specifications. First, we assessed the sensitivity of the results to the timing of the posttreatment period using event study models that allowed open enrollment 2018 to be a transition period and open enrollment 2019 to be the postcut period. The rationale of this analysis was that some advertisers were notified about the navigator funding cuts too late to alter their advertising strategy for the 2018 open enrollment period. Second, we estimated alternate models that used 2016, rather than 2015, as the first year of the analysis, because 2016 was the first year in which insurers could rely on more than a year of individual marketplace experience to inform rates and strategy.^49^ Third, we conducted additional analyses that did not include the number of insurance carriers as a control variable because recent evidence suggests that insurers are slow to modify advertising plans according to changes in competition levels.^37^ Finally, we assessed whether a single state, including Iowa, which adopted alternate Farm Bureau insurance plans during our study period, might be driving the results by systematically dropping states 1 at a time and reestimating the models. The eAppendix in the Supplement provides further details.

## Results

The final analytical sample contained 2435 counties. Table 1 shows the baseline characteristics of counties with local navigator programs (1333 counties) and counties without local navigator programs (1102 counties) during the 2015 open enrollment period. The baseline number of advertisements aired in counties with local navigator programs was 1655. Compared with counties without local programs, those with local programs had, on average, fewer private Medicare airings (mean [median; IQR] 556 [319; 104-868] vs 633 [293;104-812] airings). Counties with local navigator programs also had a larger mean (SE) population (79 930 [6801] vs 58 093 [4502] people) and a higher uninsured rate and more uninsured individuals (10 231 individuals [13.5%] vs 6668 individuals [12.0%]). The 2 groups of counties were similarly likely to be classified as rural and had similar numbers of both total airings by private sponsors and airings by private sponsors that targeted the marketplace or other non-Medicare, non-Medicaid insurance at baseline. The Figure depicts the sample counties and highlights those that were served by local navigator programs.

**Table 1.** Baseline Descriptive Statistics for Counties With Higher vs Lower Exposure to Navigator Program Cuts

Characteristic	Counties, No.		*P* value^c^
	Lower exposure (n = 1102)^a^	Higher exposure (n = 1333)^b^	
Kantar/Campaign Media Analysis Group, 2015			
Airings by private sponsors, Mean (Median; IQR)	2241 (1947; 800-3560)	2283 (1354; 406-3156)	.63
Airings by private sponsors: Private non-Medicare, non-Medicaid, Mean (Median; IQR)	1551(1146; 521-2207)	1655(986; 258-2026)	.12
Airings by private sponsors: Medicare focus, Mean (Median; IQR)	633 (319; 104-868)	556 (293; 104-812)	.006
Small Area Health Insurance Estimates data, 2015			
Population, No. of individuals	58 093	79 930	.007
Uninsured individuals, No. (%)	6668 (12.0)	10 231 (13.5)	<.001
Uninsured individuals if income is 138%-400% Federal Poverty Level, No. (%)	3084 (5.7)	4840 (6.3)	<.001
Classified as rural county per 2013 US Department of Agriculture data, No. (%)^d^	220 (20.0)	296 (22.0)	.18

^a^ Lower-exposure column displays the characteristics of low-exposure counties in 2013 or 2015 (baseline was 2015, but data on rural vs urban status were available in 2013 only).

^b^ Higher-exposure column displays the characteristics of high-exposure counties in 2013 or 2015 (baseline was 2015, but data on rural vs urban status were available in 2013 only).

^c^ Shown are the *P* values for a test of the null hypothesis that the means of the 2 groups are equal.

^d^ Rural-Urban Commuting Area codes from the US Department of Agriculture Economic Research Service,^44^ 2013, were used to define rurality. Counties with a code of 8 or 9 were classified as rural.

**Figure.**
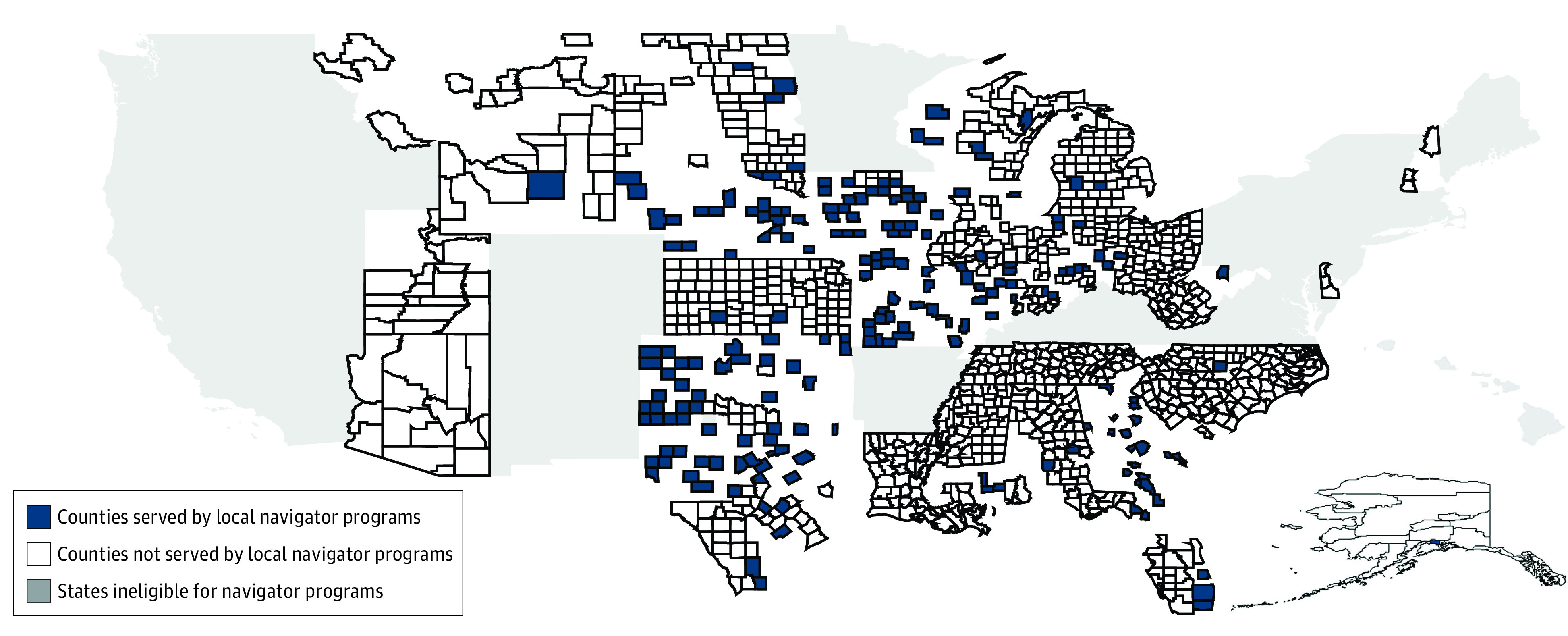
Location of Counties With Higher vs Lower Exposure to Navigator Program Cuts (ie, Counties With vs Without Local Navigator Programs in 2016) The higher-exposure counties in the analysis (shown in navy) were served by 1 or more nonstatewide (local) navigator programs in 2016. The lower-exposure counties (shown in white) were not served by local navigator programs in 2016 and, thus, were less exposed to cuts to these programs. As required by the Patient Protection and Affordable Care Act, all states have assister programs; the states shown in gray were ineligible for navigator grants but received federal funding via other mechanisms to establish alternative assister programs. Map was created by us using Stata version 16.1 (StataCorp) using data from the Centers for Medicare & Medicaid Services.

Table 2 presents the results of a difference-in-differences model of the additional change in airings associated with higher exposure to navigator program cuts, after adjustment for population and other factors. Higher exposure to funding cuts was not associated with a significant change in the volume of airings from private sponsors (point estimate, 28.8 airings per open enrollment period [95% CI, −130.1 to 160.6 airings per open enrollment period]; 1% change compared with baseline). Furthermore, greater exposure to the cuts was not associated with a significant change in marketplace or other non-Medicare, non-Medicaid airings by private sponsors or state sponsors (point estimate for private sponsors, −1.0 airings [95% CI, −92.0 to 89.9 airings]; 0.1% change from baseline). These 95% CIs rule out changes in private sector advertising volumes targeting the marketplace or other non-Medicare, non-Medicaid plans of more than 5.6%, or changes in all private sector television advertising of more than 7.0%. In the comparison case, there was no significant change in Medicare-focused airings from private sponsors, as expected given that the navigator program did not directly affect this market (point estimate, 22.4 airings [95% CI, −50.0 to 95.3 airings]; 4% change from baseline). eTable 1 in the Supplement includes further details on the model coefficients, and eTable 2, eTable 3, and eTable 4 in the Supplement present the results of further sensitivity analysis, all of which are qualitatively similar to our main findings. Substantially similar results from event study analysis are shown in eFigure 2 in the Supplement.

**Table 2.** Changes in Health Insurance Advertising Associated With Higher Exposure to Navigator Program Cuts^a^

Variable	Baseline in higher-exposure counties, mean (SE)	Difference-in-differences estimate	
		Advertisements, No. (95% CI)	*P* value
All airings by private sponsors	2282.9 (69.4)	28.8 (−130.1 to 160.6)	.67
Airings by private sponsors with Marketplace or other non-Medicaid, non-Medicare focus	1655.4 (53.5)	−1.0 (−92.0 to 89.9)	.98
Airings by private sponsors with Medicare focus	556.0 (17.6)	22.4 (−50 to 95.3)	.55

^a^ Regression models were adjusted for county population, time-invariant county-level characteristics, state-by-year secular trends, and local marketplace characteristics as noted in the text. SEs are clustered by state-treatment group.

## Discussion

Prior research^24,50,51^ has linked insurance-related advertising to increases in enrollees’ perceptions of being informed about the ACA as well as increases in shopping for and enrollment in coverage. Navigator programs, which provide outreach and one-on-one assistance, have been linked with coverage gains among marginalized groups.^13,14,25,52^ Despite the importance of these 2 forms of outreach for enrollment outcomes, few prior studies have examined how public and private sector actors conducting outreach react to one another’s efforts.

This economic evaluation tested whether private sector advertising volumes changed in response to large cuts in the navigator program, 1 of the key avenues for public sector outreach in the ACA marketplace. We were unable to detect any change in privately sponsored advertising volumes associated with the nearly 80% decrease in navigator funding implemented by the Trump administration. Furthermore, the point estimates of the change in the primary outcome of interest were small (eg, a decline of 1 advertisement annually or a 0.1% change compared with the 1655 advertisements aired at baseline). Event studies of prior trends supported the validity of the analysis, and supplemental analyses suggested the findings were robust to alternate specifications. In sum, the data do not support the hypothesis that private sector actors increased advertising to compensate for declines in navigator activity.

This evidence of a lack of responsiveness of television advertising to navigator program cuts can inform the administration of outreach in the marketplace. For example, Georgia applied for a Section 1332 waiver that would eliminate the use of the federal HealthCare.gov website for enrollment.^53^ Instead, private sector entities such as brokers, agents, and other enhanced direct enrollment entities would determine eligibility, estimate subsidies, and effectuate enrollment.^54^ As part of this change, the state would end the public sector navigator program in Georgia, under the assumption that private sector activity would compensate for its absence.

Our findings are consistent with prior evidence suggesting that private sector actors target outreach to individuals who are likely to be profitable, whereas public sector actors often use other criteria to prioritize outreach activities.^55,56^ For example, prior studies^40^ of health insurance advertising suggest private sector outreach is targeted to manage risk and maximize profit. In contrast, government-funded navigator programs target difficult-to-reach, low-income, or marginalized individuals,^23,55,56^ populations that may or may not be profitable to insurers. Indeed, 1 study^57^ found that individuals who earned between 100% and 138% of the Federal Poverty Level had the highest expenditures controlling for plan actuarial value and risk adjustment score, suggesting low-income enrollees may be less profitable to enroll than higher income enrollees.

### Limitations

This study has limitations. First, potential confounding from unobserved, concurrent changes that varied by county may have impacted the results. Second, the estimates are specific to the group of states in the sample (eg, the 33 exclusively HealthCare.gov states) and may not generalize to other states. States with state-based marketplaces fund and operate their own advertising and enrollment assistance programs.^58^ Third, although we have the most comprehensive data available on local broadcast and national cable television advertising, the data do not include advertising on local cable channels, and we were unable to observe or quantify nontelevision advertising. Fourth, the measure of advertising volume includes ads for nonmarketplace plans and non–ACA-compliant plans (eg, short-term, limited duration plans), as we were unable to identify these separately from advertisements for marketplace plans. Fifth, our findings may not represent the experience of states without county-level variation in the presence of local navigator programs (ie, states that had no local navigator programs at all, or states that had local navigator programs in every county). Sixth, although the point estimates of the change in the primary outcome of interest were small, the width of the 95% CIs imply we could not rule out changes in private sector advertising of up to 7% of the baseline level. Additionally, we were unable to assess the timing between the notification of initial navigator funding cuts and commitment deadlines for television advertising purchases.

## Conclusions

In this observational economic evaluation study of the association between television advertising and navigator funding in the ACA marketplaces, we found no association between cuts in navigator funding and changes in private sector television advertising. Advertising was not a substitute for a change in federally funded enrollment assistance. Information pathways for individuals who sought insurance are complex and channels such as privately sponsored advertising and federally funded navigator programs may not have interacted with each other. This lack of interaction can inform the design of state waivers and federal funding allocations for enrollment and outreach efforts.

## Notes

Supplement. **eAppendix.** Additional Details on Empirical Analysis **eTable 1.** Full Model with All Covariates **eTable 2.** Results of Alternate Analyses—Selectively Eliminating States from the Sample **eTable 3.** Results of Alternate Analyses—Using 2016 as Start Year of Analysis **eTable 4.** Results of Alternate Analyses—Changing Covariates **eFigure 1.** Navigator Funding Over Time **eFigure 2.** Changes in Advertising Associated with Higher Exposure to Navigator Program Cuts: Event Study Results
eReference
