# Error in Figure

**DOI:** 10.1001/jamanetworkopen.2022.36321

**Published:** 2022-09-22

In the Original Investigation titled “Association of Funding Cuts to the Patient Protection and Affordable Care Act Navigator Program With Privately Sponsored Television Advertising,”^1^ published August 1, 2022, there was an error in the Figure. The map was missing counties. This article has been corrected.^1^
