# Supplementary material for: Association of Funding Cuts to the Patient Protection and Affordable Care Act Navigator Program With Privately Sponsored Television Advertising
Source: JAMA Netw Open. 2022 Aug 1;5(8):e2224651. doi: 10.1001/jamanetworkopen.2022.24651 (PMC9344363; doi:10.1001/jamanetworkopen.2022.24651)
Supplement: Supplement. — eAppendix. Additional Details on Empirical Analysis eTable 1. Full Model with All Covariates eTable 2. Results of Alternate Analyses—Selectively Eliminating States from the Sample eTable 3. Results of Alternate Analyses—Using 2016 as Start Year of Analysis eTable 4. Results of Alternate Analyses—Changing Covariates eFigure 1. Navigator Funding Over Time eFigure 2. Changes in Advertising Associated with Higher Exposure to Navigator Program Cuts: Event Study Results eReference [file jamanetwopen-e2224651-s001.pdf]

Supplemental Online Content

Myerson R, Anderson DM, Baum LM, Fowler EF, Gollust SE, Shafer PR. Association of funding cuts to the Patient Protection and Affordable Care Act navigator program with privately sponsored television advertising. *JAMA Netw Open*. 2022;5(8):e2224651. doi:10.1001/jamanetworkopen.2022.24651

eAppendix. Additional Details on Empirical Analysis
eTable 1. Full Model with All Covariates
eTable 2. Results of Alternate Analyses—Selectively Eliminating States from the Sample
eTable 3. Results of Alternate Analyses—Using 2016 as Start Year of Analysis
eTable 4. Results of Alternate Analyses—Changing Covariates
eFigure 1. Navigator Funding Over Time
eFigure 2. Changes in Advertising Associated with Higher Exposure to Navigator Program Cuts: Event Study Results
eReference

This supplemental material has been provided by the authors to give readers additional information about their work.

© 2022 Myerson R et al. *JAMA Network Open.*

Online eAppendix 1: Additional Details on Empirical Analyses

The empirical strategy compared changes in health insurance and health care outcomes across counties within states, before versus after cuts in funding for the navigator program. We used county-level differences in prior exposure to the local navigator programs within each state for identification. Specifically, models compared changes in advertising outcomes across counties served by any of the state's local navigator programs in 2016 (higher-exposure counties) and those not served by local navigator programs in 2016 (lower-exposure counties).

The main analyses used a difference-in-difference model of the following specification:

$$y_{cst} = \alpha_c + \varphi_{st} + \delta HigherExposure_c \times I(t \geq 2018) + \beta Z_{ct} + \varepsilon_{cst} \quad (1)$$

In this model, y_{cst} represents an outcome (e.g., volume of private sector advertising) within county *c* in state *s* in year *t*; α_c is a vector of county fixed effects; and φ_{st} is a vector of state-by-year fixed effects. $HigherExposure_c$ is an indicator of whether the county was served by local (non-statewide) navigator programs in 2016 and was therefore more exposed to the subsequent cuts in program funding. $I(t \geq 2018)$ is an indicator variable that takes the value 1 starting in the post-cut period, which is Open Enrollment (OE) 2018 or later. Z_{ct} is a vector of control variables that vary across counties over time and are key determinants of advertising strategy, including: the population size of the county; marketplace characteristics including the benchmark premium, the spread between the benchmark premium and the least expensive silver plan for a single 40-year old, and the number of carriers participating in the marketplace. In alternate specifications, we excluded the number of carriers as a control. Heteroskedasticity-robust standard errors were clustered by state and exposure status of the county (more-exposed versus less exposed) to account for correlation of the error terms in regions with similar baseline exposure to the navigator program.[31] The coefficient of interest was δ, which captured changes in advertising outcomes associated with the treatment (i.e., greater exposure to cuts in the navigator program).

The validity of the analysis relied on the assumption that trends in outcomes in the two groups of counties — higher exposure and lower exposure — would have remained parallel in the absence of the funding cuts initiated under the Trump administration. While this assumption cannot be tested, a lack of pre-existing differences between trends in higher-exposure and lower-exposure counties would provide evidence of its plausibility.

To assess pre-existing differences between the trends in the two county groups, we used event study models; these models also enabled us to test for time-varying effects of funding cuts to the navigator program. These models used the following specification:

$$y_{cst} = \alpha_c + \varphi_{st} + HigherExposure_c \times \sum_{\substack{k=-3 \\ k\neq -1}}^{1} \delta_k I(t - 2018 = k) + \beta Z_{ct} + \varepsilon_{cst} \quad (2)$$

where $I(t - 2018 = k)$ is an indicator variable that takes the value 1 if year *t* is *k* years away from the start of the post-cut period (OE 2018); the omitted category was the final year of the pre-cut period (OE 2017). All other variables are defined as in model (1).

The coefficients of interest in the event study models were δ_0 and δ_1, which captured changes in outcomes associated with the treatment (i.e., greater exposure to cuts in the navigator program) in OE 2018 and 2019, respectively.

Given the late notice of changes to the navigator program, which occurred shortly prior to the start of OE 2018, advertisers might not have been able to adjust their advertisements airings for that open enrollment period. To account for this possibility, one could consider an alternate interpretation of the data in which OE 2018 is considered a transition period. In this alternate interpretation, the coefficient of interest would be δ_1, capturing changes in outcomes associated with the treatment (i.e., higher exposure to cuts in the navigator program) only in OE 2019.

To test for parallel trends across the two groups prior to funding cuts, we conducted an F-test of the joint significance of the coefficients identifying changes in the differences between high-exposure and low-exposure counties during the pre-cut period (i.e., δ_{-3} and δ_{-2}, given that the year prior to cuts is the omitted category). A non-significant result would indicate parallel trends in these counties, and thus support the validity of the analysis.

To interpret the sign of the coefficients of interest, it is helpful to know that higher-exposure counties experienced larger cuts to their navigator program funding than lower-exposure counties. For evidence of this pattern for a range of plausible assumptions, see prior work.[1]

© 2022 Myerson R et al. *JAMA Network Open.*

eTable1: Full Model with All Covariates

	Outcome: Airings by private sponsors[a]		
	All	Marketplace or other non-Medicare, non-Medicaid focus	Medicare focus
**Variables**	**(1)**	**(2)**	**(3)**
DID variable	28.78<br>(-105.8 - 163.3)	-1.024<br>(-93.85 - 91.80)	22.41<br>(-51.96 - 96.78)
Population	0.00203<br>(-0.0162 - 0.0202)	-0.00534<br>(-0.0264 - 0.0157)	0.00752<br>(0.00421 - 0.0108)
Minimum Benchmark Spread	-0.279<br>(-1.957 - 1.398)	0.387<br>(-0.899 - 1.672)	-0.635<br>(-1.272 - 0.00257)
Benchmark Rate	-0.0316<br>(-1.510 - 1.447)	0.225<br>(-0.827 - 1.278)	-0.225<br>(-0.864 - 0.414)
Number of Insurers in County	113.3<br>(31.77 - 194.7)	121.1<br>(35.37 - 206.8)	-19.68<br>(-49.56 - 10.20)
Constant	1,254<br>(-193.3 - 2,701)	852.5<br>(-721.6 - 2,427)	353.4<br>(-12.49 - 719.2)
Observations	12,173	12,173	12,173

[a] Regression models adjusted for county population, time-invariant county-level characteristics, state-by-year secular trends, and local marketplace characteristics as noted in the text. 95% confidence intervals are in parentheses.

© 2022 Myerson R et al. *JAMA Network Open.*

eTable 2: Results of Alternate Analyses – Selectively Eliminating States from the Sample

Panel A. Outcome is all airings by private sponsors

Subgroup	Baseline mean in higher-exposure counties	Difference-in-differences estimate[a]	
		No. of advertisements (95% CI).	*P*-value
All States	2282.9	28.8 (-103.1 to 160.6)	0.67
States that have both groups of counties (more-exposed and less-exposed to cuts)	2307.0	28.4 (-104.2 to 161)	0.68
Drop Alabama	2276.5	17.7 (-117.8 to 153.1)	0.80
Drop Alaska	2284.2	29.2 (-103.1 to 161.4)	0.67
Drop Arizona	2282.9	29 (-102.3 to 160.3)	0.67
Drop Delaware	2282.9	28.8 (-103.1 to 123.5)	0.67
Drop Florida	2281.0	11 (-123.5 to 145.5)	0.87
Drop Georgia	2192.5	28.5 (-103.7 to 160.7)	0.67
Drop Illinois	2302.9	43.4 (-96.3 to 183.2)	0.54
Drop Indiana	2264.9	39.6 (-102.1 to 181.3)	0.58
Drop Iowa	2378.6	28.3 (-105.3 to 161.9)	0.68
Drop Kansas	2294.3	25.9 (-110 to 161.8)	0.71
Drop Louisiana	2289.7	20.1 (-113.2 to 153.4)	0.77
Drop Maine	2278.1	29.3 (-104.2 to 162.7)	0.67
Drop Michigan	2285.1	37.6 (-99.0 to 174.2)	0.59
Drop Mississippi	2288.8	29.3 (-110.1 to 168.7)	0.68
Drop Missouri	2307.4	28.5 (-103.1 to 160.0)	0.67
Drop Montana	2317.0	30.6 (-105.8 to 167.1)	0.66
Drop Nebraska	2363.2	28.7 (-104.1 to 161.6)	0.67
Drop New Hampshire	2276.7	26.6 (-106 to 159.1)	0.69
Drop New Jersey	2228.6	-2.8 (-125.7 to 120.2)	0.97
Drop North Carolina	2267.4	24.0 (-111.7 to 159.7)	0.73
Drop North Dakota	2316.6	29.3 (-108.3 to 166.9)	0.68
Drop Ohio	2276.6	52.4 (-81.1 to 185.9)	0.44
Drop Oklahoma	2340.2	29.4 (-102.8 to 161.7)	0.66
Drop Pennsylvania	2174.0	36.5 (-99.2 to 172.3)	0.60
Drop South Carolina	2290.6	21.3 (-112.8 to 155.4)	0.76
Drop South Dakota	2262.1	30.4 (-108.6 to 169.4)	0.67
Drop Tennessee	2291.1	40.9 (-95.5 to 177.2)	0.56
Drop Texas	2157.0	77.6 (-50.5 to 205.7)	0.24
Drop Utah	2268.1	27.9 (-107.2 to 162.9)	0.69

© 2022 Myerson R et al. *JAMA Network Open.*

Drop Virginia	2299.2	-18.3 (-122.2 to 85.5)	0.73
	**Baseline mean in higher-exposure counties**	**Difference-in-differences estimate**	
		**No. of advertisements (95% CI).**	***P*-value**
Drop West Virginia	2310.8	39.6 (-98.3 to 177.5)	0.57
Drop Wisconsin	2309.7	23.7 (-112.9 to 160.3)	0.73
Drop Wyoming	2283.8	31.6 (-102.9 to 166)	0.65

[a] Regression models adjusted for county population, time-invariant county-level characteristics, state-by-year secular trends, and local marketplace characteristics as noted in the text.

Panel B. Outcome is airings by private sponsors with Marketplace or other non-Medicaid, non-Medicare focus

**Subgroup**	**Baseline mean in higher-exposure counties**	**Difference-in-differences estimate**[a]	
		**No. of advertisements (95% CI)**	***P*-value**
All States	1655.4	-1.0 (-92 to 89.9)	0.98
States that have both groups of counties (more-exposed and less-exposed to cuts)	1696.2	0.9 (-90.4 to 92.2)	0.98
Drop Alabama	1657.7	-7.5 (-101.4 to 86.5)	0.88
Drop Alaska	1656.2	-0.5 (-91.8 to 90.7)	0.99
Drop Arizona	1655.4	-0.8 (-91.5 to 89.8)	0.99
Drop Delaware	1655.4	-1.0 (-92.0 to 89.9)	0.98
Drop Florida	1663.1	2.0 (-94.2 to 98.1)	0.97
Drop Georgia	1612.9	0.1 (-91.3 to 91.6)	>0.99
Drop Illinois	1668.2	7.1 (-89.9 to 104)	0.89
Drop Indiana	1649.6	9 (-88.0 to 105.9)	0.86
Drop Iowa	1720.3	3.1 (-89 to 95.2)	0.95
Drop Kansas	1662.9	-4.3 (-98.0 to 89.4)	0.93
Drop Louisiana	1661.1	-2.0 (-95 to 91.1)	0.97
Drop Maine	1652.8	-0.9 (-93.0 to 91.1)	0.98
Drop Michigan	1657.4	5.9 (-88.2 to 100)	0.90
Drop Mississippi	1672.3	-10.1 (-104.5 to 84.4)	0.83
Drop Missouri	1667.0	-0.7 (-91.5 to 90.2)	0.99
Drop Montana	1675.5	0.1 (-94.1 to 94.2)	>0.99
Drop Nebraska	1716.0	-1.1 (-92.2 to 90.1)	0.98
Drop New Hampshire	1652.7	-2.8 (-94.2 to 88.7)	0.95
Drop New Jersey	1617.8	-18.8 (-106.5 to 68.8)	0.67
Drop North Carolina	1645.0	2.4 (-91.5 to 96.4)	0.96
Drop North Dakota	1678.0	-0.3 (-95.3 to 94.7)	>0.99

© 2022 Myerson R et al. *JAMA Network Open.*

Drop Ohio	1656.3	21.4 (-66.5 to 109.3)	0.63
Drop Oklahoma	1690.3	-1.1 (-92.1 to 90)	0.98
	**Baseline mean in higher-exposure counties**	**Difference-in-differences estimate**	
		**No. of advertisements (95% CI).**	***P*-value**
Drop Pennsylvania	1574.2	6.0 (-87.1 to 99.2)	0.90
Drop South Carolina	1659.9	-13.3 (-103.8 to 77.1)	0.77
Drop South Dakota	1622.9	-1.8 (-97.5 to 94)	0.97
Drop Tennessee	1664.3	-10.4 (-102.1 to 81.4)	0.83
Drop Texas	1529.7	22.9 (-74.0 to 119.8)	0.64
Drop Utah	1642.3	-3.5 (-96.4 to 89.4)	0.94
Drop Virginia	1671.8	-36.7 (-104.8 to 31.4)	0.30
Drop West Virginia	1678.5	0.6 (-94.5 to 95.6)	0.99
Drop Wisconsin	1670.5	7.8 (-86.9 to 102.5)	0.87
Drop Wyoming	1655.3	1.0 (-91.7 to 93.7)	0.98

[a] Regression models adjusted for county population, time-invariant county-level characteristics, state-by-year secular trends, and local marketplace characteristics as noted in the text.

Panel C. Outcome is airings by private sponsors with Medicare focus

**Subgroup**	**Baseline mean in higher-exposure counties**	**Difference-in-differences estimate[a]**	
		**No. of advertisements (95% CI).**	***P*-value**
All States	556.0	22.4 (-50.5 to 95.3)	0.55
States that have both groups of counties (more-exposed and less-exposed to cuts)	547.4	20.2 (-52.6 to 93)	0.59
Drop Alabama	547.7	18.7 (-56.8 to 94.1)	0.63
Drop Alaska	556.4	22.3 (-50.8 to 95.4)	0.55
Drop Arizona	556.0	22.4 (-50.7 to 95.6)	0.55
Drop Delaware	556.0	22.4 (-50.5 to 95.3)	0.55
Drop Florida	546.1	4.1 (-65.7 to 74)	0.91
Drop Georgia	515.5	21.0 (-51.2 to 93.3)	0.57
Drop Illinois	561.4	24.8 (-53.8 to 103.5)	0.54
Drop Indiana	553.2	15 (-62.4 to 92.3)	0.71
Drop Iowa	581.4	17.8 (-55.3 to 90.9)	0.63
Drop Kansas	559.3	22.8 (-52.6 to 98.2)	0.55
Drop Louisiana	556.7	15.4 (-57.4 to 88.1)	0.68
Drop Maine	556.2	22.7 (-51.1 to 96.5)	0.55
Drop Michigan	556.3	24.3 (-51.2 to 99.9)	0.53
Drop Mississippi	545.5	32.7 (-43.6 to 109)	0.40

© 2022 Myerson R et al. *JAMA Network Open.*

	Baseline mean in higher-exposure counties	Difference-in-differences estimate	
		**No. of advertisements (95% CI).**	***P*-value**
Drop Missouri	570.0	21.7 (-51.1 to 94.6)	0.56
Drop Montana	567.6	22.8 (-52.6 to 98.1)	0.55
Drop Nebraska	574.0	22.4 (-50.8 to 95.6)	0.55
Drop New Hampshire	553.2	22.3 (-51.1 to 95.7)	0.55
Drop New Jersey	539.0	10.4 (-60.4 to 81.2)	0.77
Drop North Carolina	550.2	15.1 (-58.6 to 88.9)	0.69
Drop North Dakota	565.6	21.8 (-54.1 to 97.8)	0.57
Drop Ohio	551.0	19.5 (-56.1 to 95.1)	0.61
Drop Oklahoma	574.0	23.1 (-50 to 96.2)	0.54
Drop Pennsylvania	527.3	23.5 (-52.7 to 99.7)	0.55
Drop South Carolina	558.8	27.0 (-47.8 to 101.8)	0.48
Drop South Dakota	565.5	24.4 (-52.5 to 101.4)	0.53
Drop Tennessee	554.8	43.6 (-24.2 to 111.3)	0.21
Drop Texas	550.2	49.2 (-19.3 to 117.8)	0.16
Drop Utah	554.9	24.0 (-50.6 to 98.7)	0.53
Drop Virginia	555.2	21.6 (-54.4 to 97.5)	0.58
Drop West Virginia	561.4	31.5 (-44.3 to 107.3)	0.42
Drop Wisconsin	570.9	7.5 (-62.9 to 77.9)	0.83
Drop Wyoming	557.0	22.0 (-52.2 to 96.1)	0.56

[a] Regression models adjusted for county population, time-invariant county-level characteristics, state-by-year secular trends, and local marketplace characteristics as noted in the text.

© 2022 Myerson R et al. *JAMA Network Open.*

eTable 3: Results of Alternate Analyses – Using 2016 as Start Year of Analysis

Outcome	Baseline mean in higher-exposure counties	Difference-in-differences estimate[a]	
		No. of advertisements (95% CI)	*P*-value
All airings by private sponsors	1825.6	11.8 (-89.5 to 113.12)	0.82
Airings by private sponsors with Marketplace or other non-Medicaid, non-Medicare focus	961.1	-33.1 (-89.3 to 23.2)	0.25
Airings by private sponsors with Medicare focus	739.2	35.4 (-34.1 to 105.0)	0.32

[a] Regression models adjusted for county population, time-invariant county-level characteristics, state-by-year secular trends, and local marketplace characteristics as noted in the text.

© 2022 Myerson R et al. *JAMA Network Open.*

eTable 4: Results of Alternate Analyses – Changing Covariates

Outcome	Baseline mean in higher-exposure counties	Difference-in-differences estimate[a]	
		No. of advertisements (95% CI).	*P*-value
All airings by private sponsors	2282.9	29.0 (-107.2 to 165.3)	0.68
Airings by private sponsors with Marketplace or other non-Medicaid, non-Medicare focus	1655.4	-0.75 (-95.9 to 94.4)	0.99
Airings by private sponsors with Medicare focus	556.0	22.4 (-50.1 to 94.8)	0.55

[a] Regression models adjusted for county population, time-invariant county-level characteristics, state-by-year secular trends, and local marketplace characteristics as noted in the text with the exception of the number of insurance carriers, which was not included as a control variable in this alternate specification.

© 2022 Myerson R et al. *JAMA Network Open.*

eFigure 1: Navigator Funding over Time

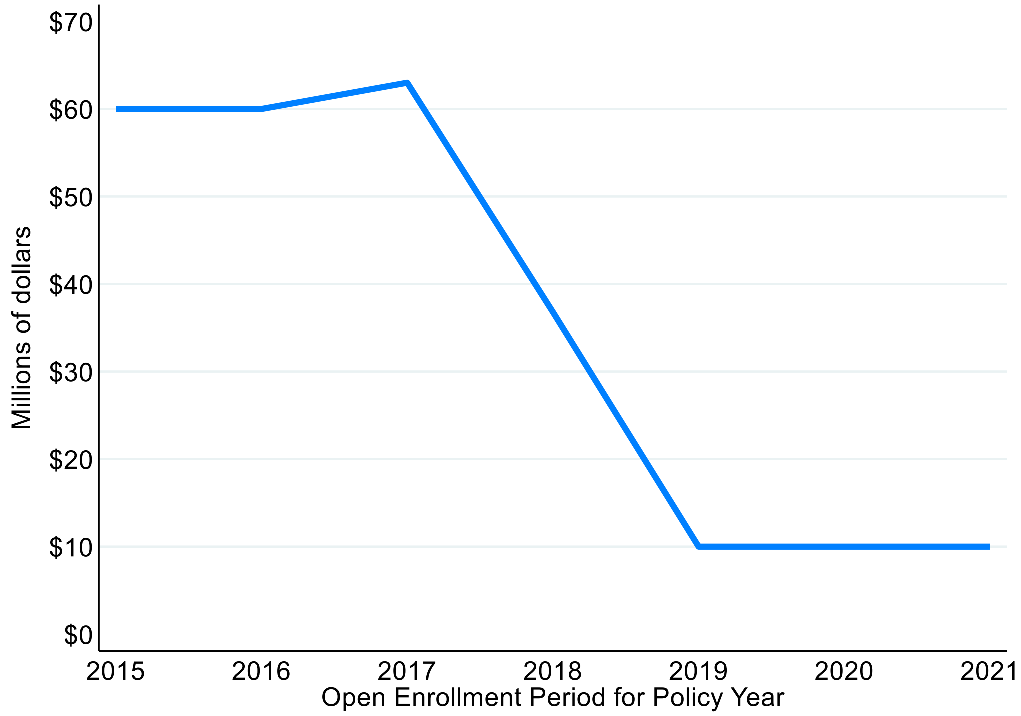

© 2022 Myerson R et al. *JAMA Network Open.*

eFigure 2: Changes in Advertising Associated with Higher Exposure to Navigator Program Cuts: Event Study Results

A. All airings by private sponsors

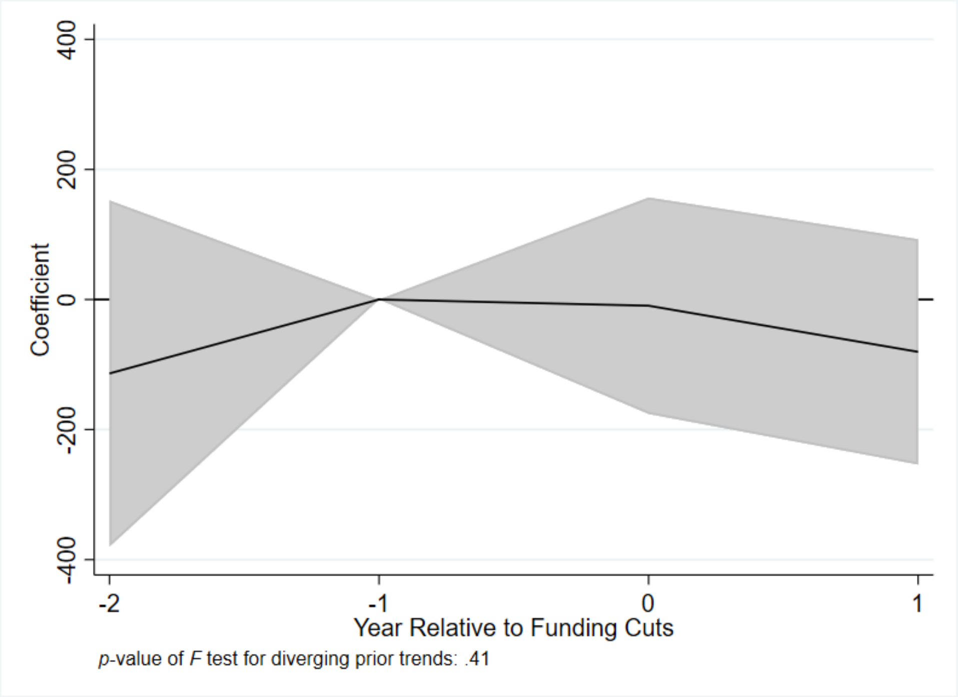

B. Airings by private sponsors with marketplace or other non-Medicaid, non-Medicare focus

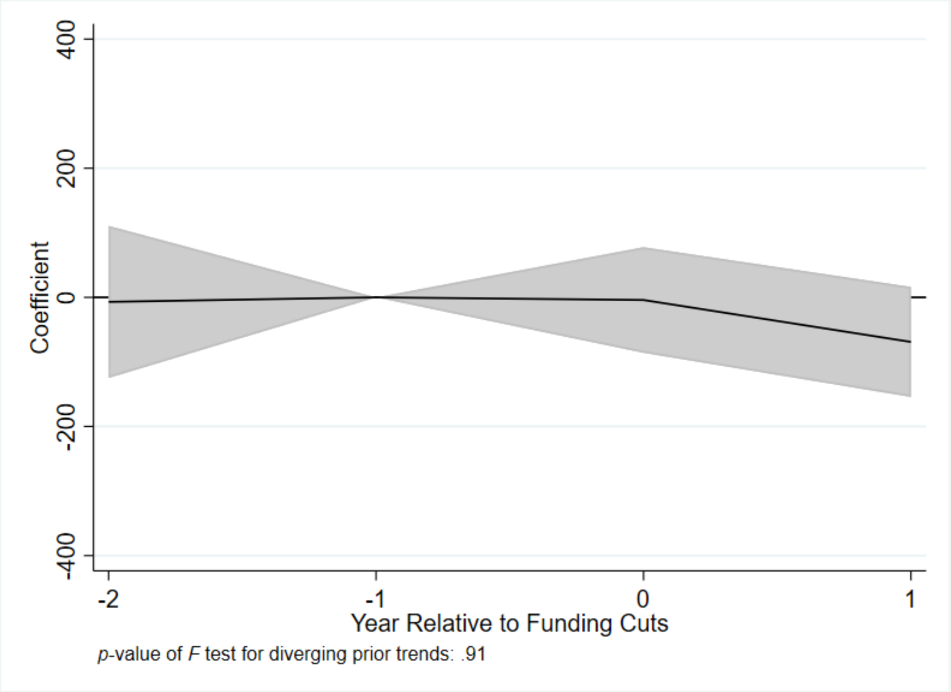

© 2022 Myerson R et al. *JAMA Network Open.*

C. Airings by private sponsors with a Medicare focus

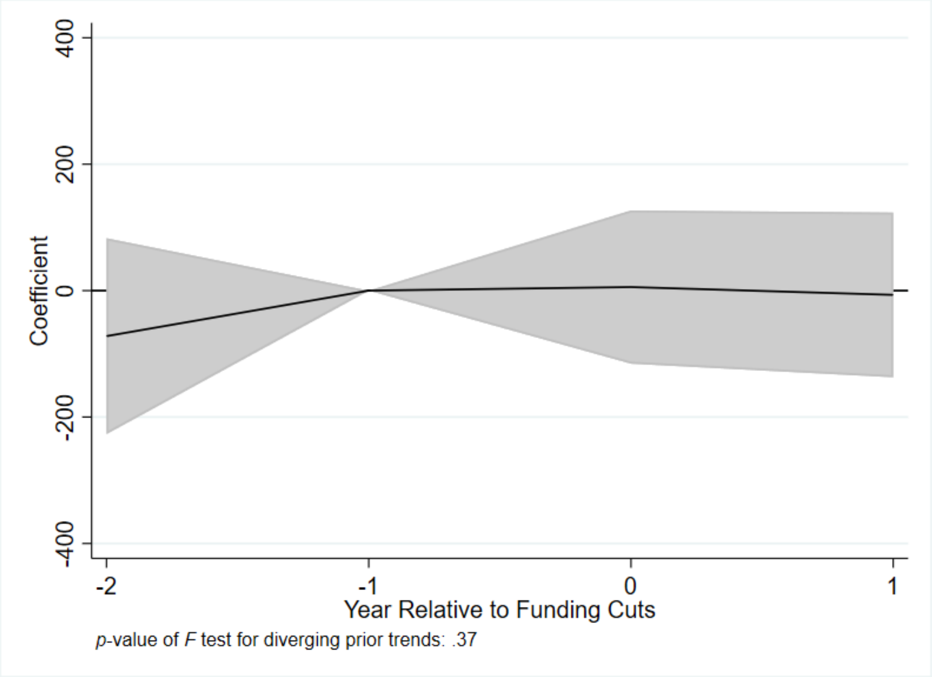

NOTE: Dotted lines are 95% confidence intervals.

© 2022 Myerson R et al. *JAMA Network Open.*

eReferences

1. Myerson R, Li H. Information Gaps and Health Insurance Enrollment: Evidence from the Affordable Care Act Navigator Programs. *Am J Health Econ*. Forthcoming. http://dx.doi.org/10.2139/ssrn.3966511

© 2022 Myerson R et al. *JAMA Network Open.*
